# Asymmetric and time-frequency co-movements among innovation-themed investments and carbon emission efficiency: Thematic investing and hedging opportunities

**DOI:** 10.1371/journal.pone.0293929

**Published:** 2024-02-29

**Authors:** Chunhui Huo, Paulo Ferreira, Inzamam Ul Haq

**Affiliations:** 1 Faculty of Economics, Asia-Australia Business College, Liaoning University, 110036, Shenyang, China; 2 VALORIZA—Research Center for Endogenous Resource Valorization, Portalegre, Portugal; 3 Department of Economic Sciences and Organizations, Polytechnic Institute of Portalegre, Portalegre, Portugal; 4 Business School, Liaoning University, 110036, Shenyang, China; Universiti Malaya, MALAYSIA

## Abstract

This study is aimed at investigating the asymmetric and time-frequency co-movements and the hedge or safe-haven properties of carbon efficient indices, the MSCI ACWI Sustainable Impact, and MSCI World EGS indices, in relation to technology and innovation-themed investments. In doing so, the ADCC-GJR-GARCH and wavelet coherence techniques are applied to a daily return series ranging from January 2019 to January 2023. Findings of the ADCC-GJR-GARCH model show negative and insignificant asymmetric linkage among underlying indices during the sample period. The S&P 500 carbon efficient index (CEI) acts as a strong hedge or safe-haven for technology and innovation-themed indices during tranquil and tumultuous periods. The MSCI ACWI Sustainable Impact, MSCI World EGS, and carbon efficient indices except for S&P 500 CEI exhibit weak hedge or safe-haven attributes. Wavelet coherence reveals negative (positive) co-movements between the thematic and carbon efficient indices in short-term (medium-term and long-term) horizons with consistent leading behavior of thematic indices to carbon efficient indices outcomes. It justifies the presence of short-lived hedging or safe-haven characteristics in the thematic domain for investors. These strong and weak hedge or safe-haven characteristics of low carbon and sustainability indices reveal that adding low carbon efficient and sustainable investments to a portfolio result in considerable diversification benefits for investors who tend to take minimal risk in both tranquil and tumultuous periods. The current findings imply that financial institutions, thematic investing companies, and governments need to encourage carbon efficient technology transfer and innovation-themed investments by increasing the fund allocations in underlying asset classes. Policy-making and regulatory bodies can encourage investors to make carbon-efficient and thematic investments and companies to issue carbon-efficient stocks or investments to safeguard social and economic risks during fragile periods. These investments can offer greater opportunities to combat the intensity of economic shocks on portfolios for responsible or sustainable investors.

## 1. Introduction

According to the US National Oceanic and Atmospheric Administration (NOAA), by 2022, atmospheric carbon emissions had reached a level 50 percent higher than that in the preindustrial era [1]. Carbon dioxide (CO_2_) reached a level of approximately 36.6 billion tons per year in 2022 [2]. Noticeably, CO_2_ is the most important contributor to climate change and global warming, evidenced by the fact that the temperature has risen by more than 1°C to 1.2°C since the preindustrial era began [3,4]. Considering the severe consequences of carbon emissions on human survival and the sustainability of the planet Earth [5], the United Nations also named climate change a ‘defining crisis of our time’ [6]. In the current global environmental crisis, carbon emissions efficiency has become a key global concern.

Mitigation of the adverse consequences of global climate change, which has drawn substantial attention across the globe, depends largely on the reduction of CO_2_ emissions [7]. In finance, the phrase carbon emissions efficiency relates to mitigating atmospheric CO_2_ through the promotion of investment inflow and capital allocation in carbon efficient and sustainable investments [4,8]. Notably, low carbon investments ensure the meeting of long-term ecological protection goals, which are important to short-term human interests and economic benefits [9]. At the UN Climate Change Conference (COP26), countries made significant commitments to reducing methane emissions, stopping and reversing forest loss, aligning the financial sector with the net-zero goal by 2050, hastening the phase-out of coal, ceasing production of the internal combustion engine, and stopping the foreign financing of fossil fuels [10]. Although there has been significant progress achieved on many fronts, national climate and financing commitments have fallen far short of what is needed to address global climate risk [10]. At present, major carbon emissions emitters need to upgrade the emission reduction targets of 2030 to align with the net-zero emissions targets, taking Finland as an example [11]. To achieve the goals set in the Paris Climate Change Agreement by 2050, a serious global climate change movement will need to raise approximately US $3.5 trillion from the energy sectors every year from 2020 to 2050, totaling approximately US $110 trillion [4]. Therefore, the investment flow and fund allocation toward decarbonization [12] and carbon-washing [13] need to be accelerated to make progress toward net-zero emissions targets and develop greater economic, social and environmental prospects.

Meanwhile, thematic investing has been evolving recently. Thematic investing is characterized as a top-down investment approach that capitalizes on opportunities created by macroeconomic, geopolitical and technological trends [14]. These are not short-term swings but rather long-term, structural, transformative shifts. These thematic shifts touch every part of people’s lives, including affecting the growing digital economy, smart cities and the cybersecurity revolution, fintech innovation and the development of disruptive and autonomous technology innovation [15]. Thematic investing is sometimes defined as impact investment and revolves around the broad movement of social financial practice aimed at transforming the investment paradigm and global economies [16]. For example, structural economic changes, such as shifting demographics and social change, technological breakthroughs, scientific discoveries, climate change and resource scarcity, are evolving at an ever-faster pace [13]. The demand for technologically advanced and environmentally sound technologies and infrastructure has become a necessity due to climate change and rapid urbanization [11,17,18]. In the current scenario, in contrast to socially responsible and sustainable investment, innovation-themed or impact investing involves initiating investments in innovative organizations to tackle societal issues [19], making such investments a relative asset class for carbon efficiency development. Despite the central role played by digitalization and technological innovation in economic, financial and social development [20], the involvement of these two factors in carbon footprints cannot be ignored [21]. The continuous increase in CO_2_ emissions results from an increase in the global energy demand associated with growth in global trade, industrial growth and the digital transformation [7,22,23]. This reveals the limited environmental benefits of transformation and industrial growth. Moreover, the policies and investment decisions pertaining to innovation and digitalization in the coming decades regarding robotics, digital economy, fintech, cybersecurity, smart cities, autonomous technology, and industrial innovation can exert a tremendous influence on the carbon emissions curve. Therefore, researching the ways that thematic investments impact carbon emissions efficiency is a timely strategy.

Previous findings indicate that digitalization [5], technological innovation [20], financial [20], economic [24] and industrial growth [7] are all major contributing factors to carbon emissions. To the best of our knowledge, this study is the first of its nature to explore the dynamic relationship among innovation and technology-based thematic investing, carbon emissions efficiency time and frequency domains. Considering Gupta and Sharma [17], it is important for investors to understand these shifting trends toward a carbon-efficient economy and to take economic, environmental and social aspects into consideration as they construct portfolios and make decisions around future strategies. This study is focused on the technology and innovation-themed indices from the MSCI-family and the low carbon or carbon efficient indices, as well as the major world sustainable impact and the EGS indices. The MSCI-family has introduced various global thematic indices, but we account for eight MSCI ACWI thematic indices that are more related to the technology, innovation, and digitalization process, as they are more aligned with the motivations underlying this study. These indices are as follows: the Autonomous Technology & Industrial Innovation Index (ATIII), Cybersecurity Index (CSI), Digital Economy Index (DEI), Fintech Innovation Index (FII), Innovation Index (INI), Next Generation internet Innovation Index (NGIII), Robotics Index (RBI) and Smart Cities Index (SCI). Moreover, the MSCI World Low Carbon Leaders Index (WLCLI), MSCI World Low Carbon Target Index (WLCTI), S&P 500 Carbon Efficient Index (S&P500CEI) and S&P Global LargeMidCap Carbon Efficient Index (S&PGLMCEI) are used as measures of global carbon efficiency. Additionally, we included the MSCI ACWI Sustainable Impact Index (SII) and World EGS Leader Index (WEGSLI) as proxies for world sustainable impact and for sustainability and environmental, governance, and social (EGS) performance, respectively.

Employing the ADCC-GJR-GARCH technique, we determine the asymmetrical nature of the volatility of all innovation-specific and carbon efficient index price returns during the health crisis, suggesting that negative shocks (bad news) have a greater impact on volatility than positive events (good news). The S&P 500 Carbon Efficient Index shows a persistent and stronger negative conditional correlation across global low carbon indices, whereas the MSCI World Low Carbon Leaders, MSCI World Low Carbon Target, S&P Global LargeMidCap Carbon Efficient, MSCI ACWI Sustainable Impact and World EGS Leader Indices show a negative but nonsignificant association during both tranquil and tumultuous periods, i.e., COVID-19. We find that S&P 500 Carbon Efficient Index offers strong hedge or safe-haven characteristics through thematic stocks of varying properties during a global health crisis. The remaining decarbonized assets serve as weak hedge or safe-haven investments. The analysis of wavelet coherence reveals negative or out-phase (positive or in-phase) co-movement between the thematic and carbon efficient indices in short-term (medium-term and long-term) investment horizons, suggesting the presence of short-lived hedging or safe-haven characteristics in the thematic domain for investors in carbon efficient investments. In addition, thematic indices play a consistent leading role in the outcomes of carbon efficient indices in medium-term and long-term investment horizons. These strong safe-haven characteristics of low-carbon investments for multiple thematic assets reveal the sizable hedging potential of carbonwashing investments, particularly for responsible investors who tend to take fewer risks in periods of economic stress and turbulence. As an asset class, the role of thematic investment in ensuring carbon efficiency shows great promise. Nevertheless, carbon-efficient financial assets have the capacity to withstand economic shocks and give risk-averse investors the chance to add a variety of hedge or safe-haven assets to their asset portfolios to reduce portfolio risks.

Our study contributes to the literature in following ways. Using the Asymmetric Dynamic Conditional Correlations (ADCC)-Glosten, Jagannathan and Runkle (GJR-GARCH) in conjunction with wavelet coherence techniques to examine whether carbon efficient investment options stipulate hedge or safe-haven characteristics in the world thematic stock market during the COVID-19 crisis, we add to the literature that examines the hedge and safe-haven properties of several EGS and sustainable financial assets [25–32]. Our results encourage investors to consider carbon-efficient investments due to their strong (weak) hedging or safe-haven characteristics in the short run (long run) to counteract the intensity of economic shocks on thematic asset portfolios during both tranquil and tumultuous periods. Moreover, we find that decarbonized assets not only address environmental-focused vulnerabilities, which is in line with previous studies [8,13,33,34], but they also contribute to the mainstream stocks and thematic stock markets by providing extensive support to institutional and individual investors in realigning their portfolios according to these rapidly changing dynamics.

The remainder of the paper is structured as follows: Section 2 discusses the hedging or safe-haven literature on sustainable and EGS investments; Section 3 presents the data and preliminary analysis; Section 4 explains the research methods; Section 5 presents and discusses the empirical findings in relation to previous studies; and Section 6 concludes the paper.

## 2. Literature review

Many studies have been conducted to examine the hedging or safe-haven of different financial assets, including EGS, sustainable and low carbon efficient investments, during turbulent times, such as the global health crisis. For instance, socially responsible and low carbon investing encourages financial growth and sustainable development and acts as a safe investment vehicle in emerging economies [27]. By way of these investments, investors can gain social, environmental, and economic benefits through diversification and hedging. Examining the tail dependence between digital currency (bitcoin) and sustainable or green investments, Naeem and Karim [26] reported dynamic dependence between these factors, employing a time-varying optimal copula and finding evidence of time-varying hedging characteristics. Notably, the hedging effectiveness and hedge ratios were lower for WEGSLI than for other green assets. Similarly, Goodell, Corbet [28] found that green economy and fintech indices have potential benefits for investors in terms of hedging digital currency’s (Bitcoin) risk and the environmental consequences of the cryptocurrency market. Koçak, Bulut [34] investigated the impact of COVID-19 cases, market volatility (VIX), economic policy uncertainty, oil prices and government response on the S&P 500 CEI using Fourier’s approach, finding a positive impact of COVID-19 and oil prices on low carbon company stocks. This demonstrated the hedging or safe-haven potential of the S&P 500 CEI as well as its resistance to the health crisis. Olofsson, Råholm [30] studied the comparative hedging or diversification potential among conventional, ethical and unethical investments using the Bayesian Markov-switching generalized autoregressive conditional heteroscedasticity model, reporting that ethical investments offer a lower portfolio beta for investors and that WEGSLI returns can serve as potential alternatives for hedging. Fareed, Abbas [29] studied the bivariate and multivariate rolling window correlations between the S&P 500 CEI and COVID-19 cases, finding a positive linkage between those variables. Moreover, the existence of safe-haven properties of the S&P 500 CEI across both bivariate and multivariate settings suggests its hedging or diversification opportunities during COVID-19. Kanamura [35] examined the relationship between energy and EGS indices, particularly the WEGSLI, and reported heterogeneous conditional correlations over time, suggesting a time-varying or limited hedging ability as well as a consistent social and governance value creation. Examining the safe-haven properties of EGS indices, i.e., WEGSLI, through a wavelet coherence framework during the global health crisis, Rubbaniy, Khalid [31] showed that the safe-haven properties are limited to the short-term investment horizon, and that they fail to act as a safe-haven against the CBOE VIX. Specifically, Piserà and Chiappini [32] investigated the hedge or safe-haven opportunities of EGS indices nationwide in China using multivariate GARCH models, i.e., dynamic conditional correlation, constant conditional correlation and varying conditional correlation. The majority of EGS indices exhibited safe-haven properties during the turmoil period of COVID-19, suggesting the superiority of environmental, social and governance investments over cryptocurrency and commodity markets [32]. Constructing a news-based index on the basis of pure-play and MSCI decarbonized indices, Apel, Betzer [36] found that short-term transition volatility has a significant impact on portfolio returns, and so a portfolio mix that adds a more diverse set of assets that are environmental performance specific may be sought during the course of hedging transition risk. Of similar importance, MSCI SII and other green bonds can offer diversification benefits through effective allocation and portfolio selection, although a higher carbon intensity fails to predict social, governance and environmental objectives [37]. Using an ADCC-GARCH model, Nakajima, Hamori [38] found [39] heterogeneous asymmetric conditional correlation between green indices, including the MSCI world EGS index and sustainability index, indicating the heterogeneous hedging or diversification potential of different green indices. Using a copula approach, Nakajima, Hamori [39] showed that sustainable and responsible investments can offer diversification benefits for renewable energy companies’ stock portfolios. Similarly, Rao, Gupta [40] revisited the interconnectedness of the financial market during COVID-19 and found that Bitcoin and gold are somewhat unreliable hedging instruments under extreme market and economic conditions; however, green bonds possess diversification benefits across normal and turbulent periods [40]. Noticeably, Sharma, Sarker [41] documented that conventional and green investments have long-term bidirectional causality and that identical risk-adjusted returns for both investments imply that investors may shift from conventional to green investments without sacrificing financial gains, particularly during opportunities involving increased funds flow in green investments [41].

Zhang, He [42] examined the connectedness among sustainability-related financial indices, i.e., the world EGS, renewable energy stock, green bonds indices and carbon emission futures. They applied DCC-GARCH-based dynamic connectedness and DCC-GARCH with t-copula techniques and found that carbon emission futures serve as net volatility transmitters to sustainability-related indices where the hedging power of sustainability-related investments remains short-lived. Similarly, applying the wavelet-based quantile-on-quantile technique, Pang Pang, Zhu [8] studied the impact of green finance on carbon efficiency considering both bullish and bearish market sentiments. They found that green finance has a positive impact on carbon efficiency in a bullish trend but a negative effect in bearish moments, suggesting that green finance does not represent a consistent blessing for carbon efficiency and green technology. Studying the relationship between financial development and energy consumption, Chiu and Lee [43] concluded that financial development across multiple sectors fosters energy consumption, whereas environment-focused financial development lowers energy consumption, which has a significant impact on carbon emissions. In a similar vein, Shahzad, Sengupta [44] reported that the carbon emission future level is reactive to extreme movement that occurs in natural gas and oil due to their direct contribution to environmental degradation and carbon emissions.

Previous research has also been conducted to explore the impact of domestic material on multiple greenhouse gas emissions. For instance, using the Fourier function approach, Alola and Adebayo [45] reported that domestic material consumption (DMC) across sectors (agriculture, industrial, waste management) affects greenhouse gas emissions heterogeneously. In particular, metallic ores DMC (biomass and fossil fuels) spur (mitigate) greenhouse gas emissions in the long run. Likewise, Jahanger, Hossain [46] found that transportation services, tourism and economic growth accelerate carbon emissions, whereas globalization insignificantly reduces carbon emissions, revealing a dire need to strengthen the service industry through the tightening of regulations and eco-friendly resource use. In a similar vein, utilizing wavelet coherence approaches, Adebayo et al. [47] documented that economic growth and natural gas energy consumption (hydro energy consumption) undergo positive (negative) co-movement with CO_2_ emissions in the long run. It has been observed that social economic conditions, economic growth and financial development are degrading environmental quality in terms of high carbon and ecological footprints due to their association with high energy consumption and increased income [48].

Due to rapid urbanization and climate change, the demand for technologically advanced and environmentally sound infrastructure is increasing [17]. In recent past, the expansion of green financial assets has stimulated investments in renewable energy and reduced the vulnerability of environmental pollution and carbon emissions [49]. In addition, social impact finance can foster economic and financial stability by promoting investments with social goals and nonspeculative financial returns [50].

The role played by technology and innovation-themed investments remains unexplored in the literature. Conventional financial markets have failed to hedge all climate-related risks, focusing on green projects and divestment from high carbon emission investments or companies [51]. Discussing future research avenues, Koçak, Bulut [34] emphasized exploring the EGS index family members in other industries, which could provide fresh evidence on carbon efficient and alternative business models. This also helps to create optimal investment portfolios and implement sustainable development plans, especially for industries such as the social transformation industry, which require investments with predictable returns and low carbon emissions. Consequently, low carbon investments can also facilitate access to the large amount of capital necessary for the transition to cleaner energy and to the objective of net zero emissions by 2050 of governments around the world [52]. Therefore, the hedging or safe-haven role of decarbonized investments needs to be explored further.

In recent years, a growing body of literature has addressed the relationships between different asset classes, regions, sectors and styles and carbon efficiency. However, knowledge of the possible impact of thematic investing on carbon efficiency is still in its infancy. We explore the notion of themes as an additional investment dimension and the dynamic conditional correlation between thematic investing and carbon efficiency under multivariate settings. This correlation has rarely been examined.

## 3. Data and preliminary analysis

### 3.1. Data and sources

This research is aimed at exploring the dynamic asymmetric correlation between major thematic and major low carbon or carbon efficient indices since these indices could play key roles in technology transfer and digitalization globally. We use the daily closing prices for the eight thematic MSCI ACWI IMI indices: ATIII, CSI, DEI, FII, INI, NGIII, RBI and SCI. As measures of global carbon efficiency and environmental performance, we consider four low carbon or carbon efficient indices, i.e., the MSCI World Low Carbon Leaders Index (WLCLI), the MSCI World Low Carbon Target Index (WLCTI), the S&P 500 Carbon Efficient Index (S&P500CEI) and the S&P Global LargeMidCap Carbon Efficient Index (S&PGLMCEI), as well as two other environmentally/socially responsible indices, the MSCI ACWI Sustainable Impact Index (SII) and the World EGS Leader Index (WEGSLI). The sample covers the period from 10 January 2019 to 10 January 2023, and the data were sourced from the official websites of S&P Global https://www.spglobal.com/spdji/en/ and MSCI https://www.msci.com/our-solutions/indexes.

The key motivation for considering the current data is to highlight the possible dynamic asymmetric features when comparing tranquil and turbulent periods, which could provide a better understanding of the changes that occurred during the COVID-19 crisis. The daily data for thematic indices were available starting in 2019, which also restricts the coverage of the overall dataset. Based on the different daily prices, the daily returns were calculated as *Rt* = *ln*(*pt*)-*ln*(*pt*-1); i.e., they were calculated using the first log differences of the closing prices, and they represented a total of 1043 observations.

### 3.2. Preliminary analysis

**Fig 1:**
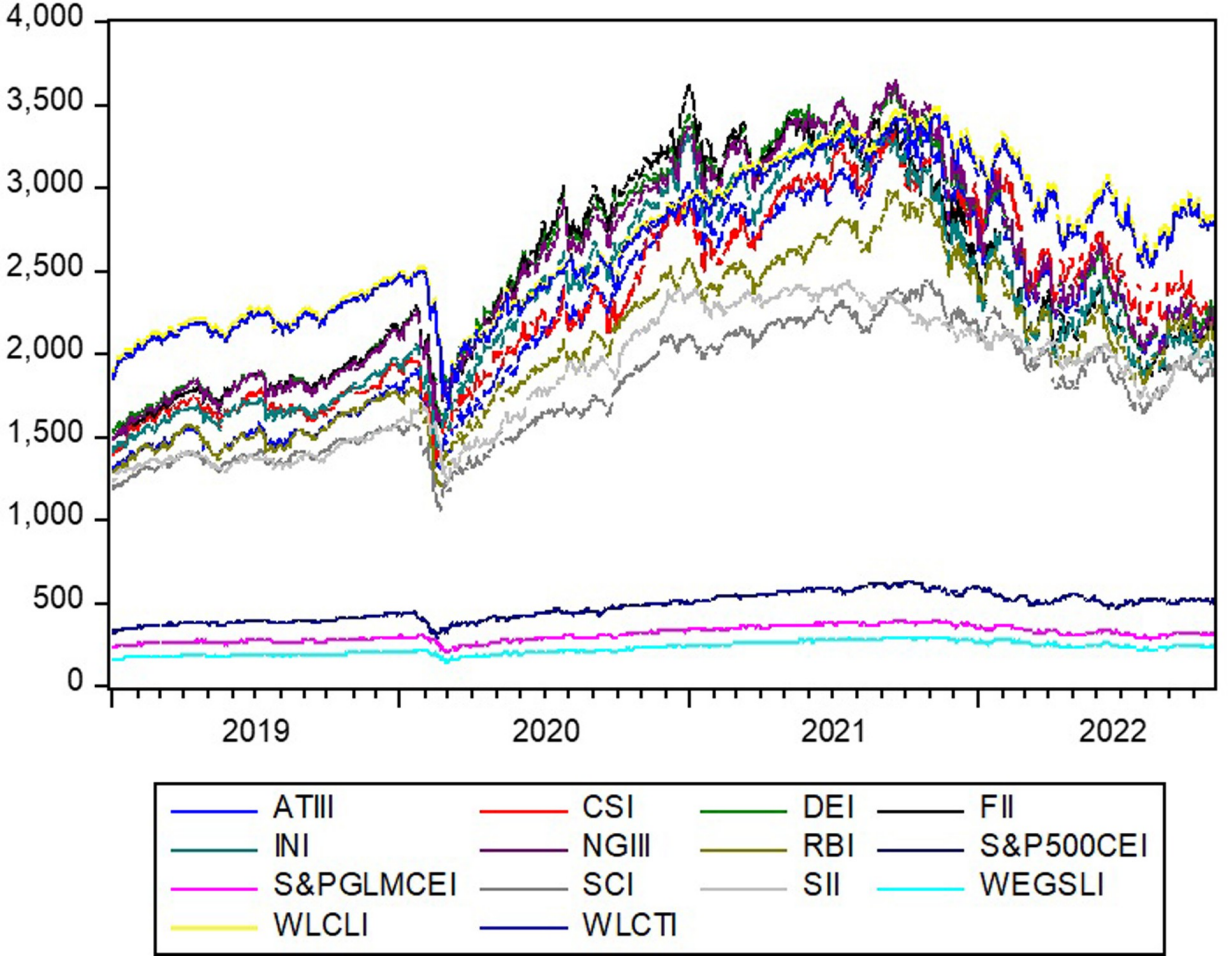
Evolution of thematic and carbon efficiency indices.

Fig 1 presents the evolution of prices, showing a common pattern for all thematic and low carbon/energy efficient indices. The plot indicates that for all the indices, there is a notable decrease in prices, suggesting that the COVID-19 outbreak contributed to a decrease in prices during the last quarter of 2019. Similar behavior regarding the relative financial asset class has been reported in research covering the COVID-19 pandemic period [20]. The abrupt decline in prices was due to investment inflow, the loss of investor trust, and risk-averse behavior during the global health crisis (GHC). Following a subsequent abrupt decline, prices for all indices showed a rising trend in 2020. It is reported that enormous capital inflow injections into these classes fostered price and overall market capitalization; for example, FinTech investments increased from $87 billion in the first quarter of 2020 to $98 billion in the first quarter of 2021 [20]. Another explanation of the upward trend that followed 2020 and served as a turning point for all EGS investments in terms of social aspects suggests that the financial crisis triggered by COVID-19 led to a change in investor perception regarding social aspects [53]. Thus, the increase in investor demand, investment inflow, and investor perception explains these patterns. Later, prices came to follow a consistent descending trend, possibly due to slower global economic growth, lower inflation, and ongoing monetary tightening.

**Fig 2:**
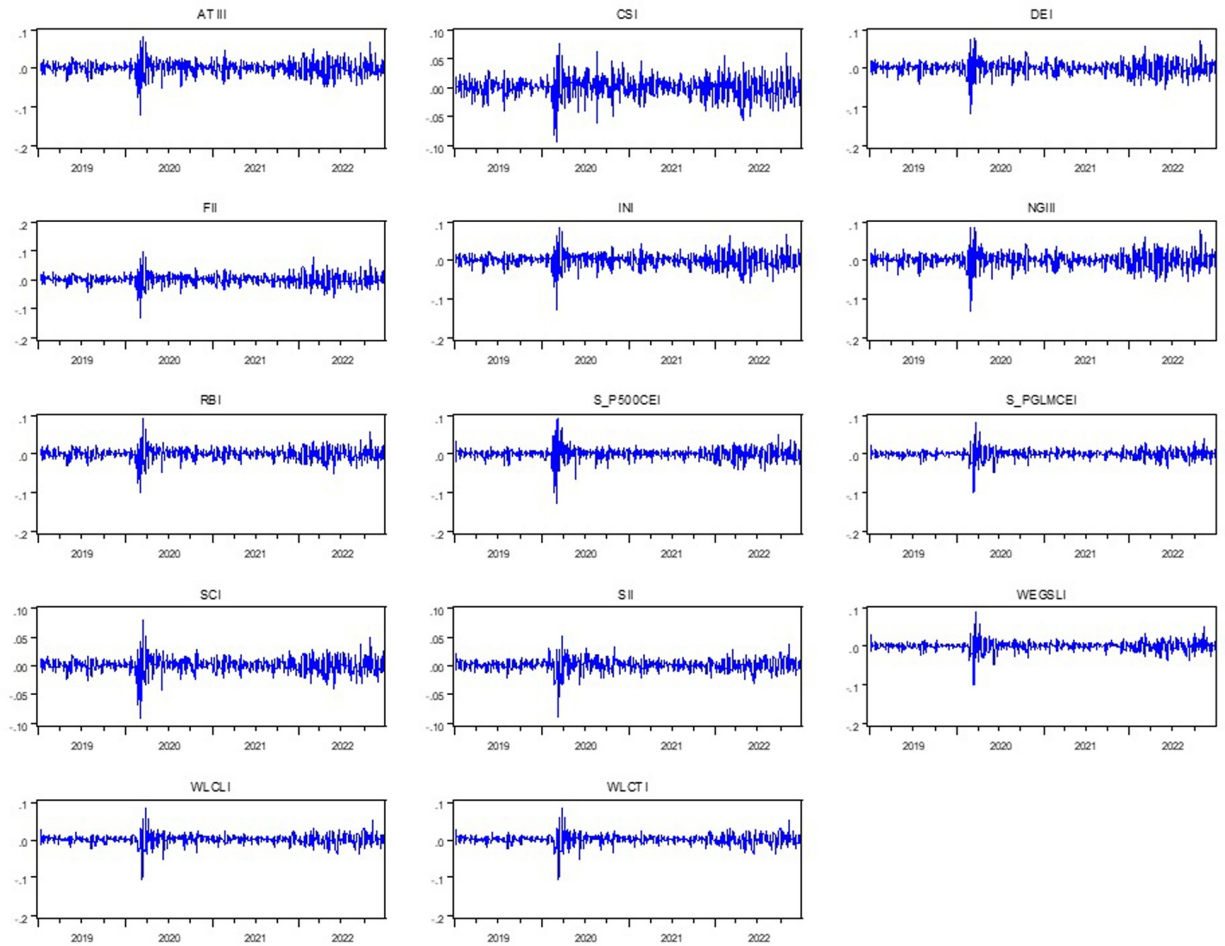
Daily differenced returns.

Fig 2 shows the evolution of daily return series for the indices under analysis, revealing an abrupt change that occurred at the beginning of the GHC. Additionally, compared to the tranquil phase, the fat tails and volatility clustering are larger in the turmoil phase, which implies that market volatility and daily price return dynamics were notably different before and after the GHC. The time-varying price returns displayed in Fig 2 could also indicate the existence of volatility clustering and fat tails, which could suggest some possible nonlinear process.

Table 1 reports the descriptive statistics for the daily returns, and Table 1 presents similar statistics for the price series. In general, the mean returns were positive for all indices. Among thematic (carbon efficient) returns, FII (WLCTI) demonstrated the highest average returns, while the lowest average returns were those of NGIII (S&P500CEI). Thematic returns were more volatile in low carbon indices, with INI (WEGSLI) showing the highest (lowest) standard deviation of 0.01758 (0.00968). Regarding skewness and kurtosis values, all of the return series present leptokurtic levels and are skewed left (i.e., the left tail was longer). The Jarque-Bera test confirms that these returns violate the properties of normal distribution, and finally, the augmented Dickey–Fuller (ADF) shows that return series were stationary during the period under analysis.

**Table 1:** Descriptive statistics.

Variables	M	Max.	Min.	SD.	Skew.	Kurt.	JB	ADF
ATIII	0.00038	0.08502	-0.12511	0.01414	-0.65004	9.23703	1767.35401	-31.17002
CSI	0.00040	0.07621	-0.09712	0.01753	-0.40201	6.47304	553.13202	-36.69002
DEI	0.00046	0.07912	-0.12121	0.01700	-0.54904	9.02403	1632.54502	-34.70004
FII	0.00048	0.10022	-0.13312	0.01217	-0.40201	9.32204	1768.25702	-31.71002
INI	0.00040	0.08513	-0.12821	0.01758	-0.55303	8.58303	1410.43801	-34.48003
NGIII	0.00037	0.08621	-0.13322	0.01593	-0.50601	8.89404	1557.01901	-34.39004
RBI	0.00038	0.09113	-0.10111	0.01645	-0.64505	10.76102	2694.94802	-32.61004
SCI	0.00044	0.08121	-0.09322	0.01660	-0.83704	13.00103	4476.68203	-35.78003
S&P 500 CEI	0.00028	0.09213	-0.13111	0.01208	-0.79603	16.11302	7582.52204	-34.61005
S&PGLMCEI	0.00041	0.08323	-0.10222	0.01213	-1.13802	18.15703	10209.44002	-34.45005
SII	0.00038	0.05213	-0.08911	0.01216	-0.92605	13.11502	4595.79801	-34.38004
WEGSLI	0.00040	0.08622	-0.10212	0.00968	-1.00204	17.66905	9526.15002	-29.18005
WLCLI	0.00046	0.08511	-0.10412	0.01170	-1.0840	17.62004	9493.19602	-33.52003
WLCTI	0.00048	0.08322	-0.10314	0.01460	-1.04301	17.07801	8801.9630	-39.44002

**Fig 3:**
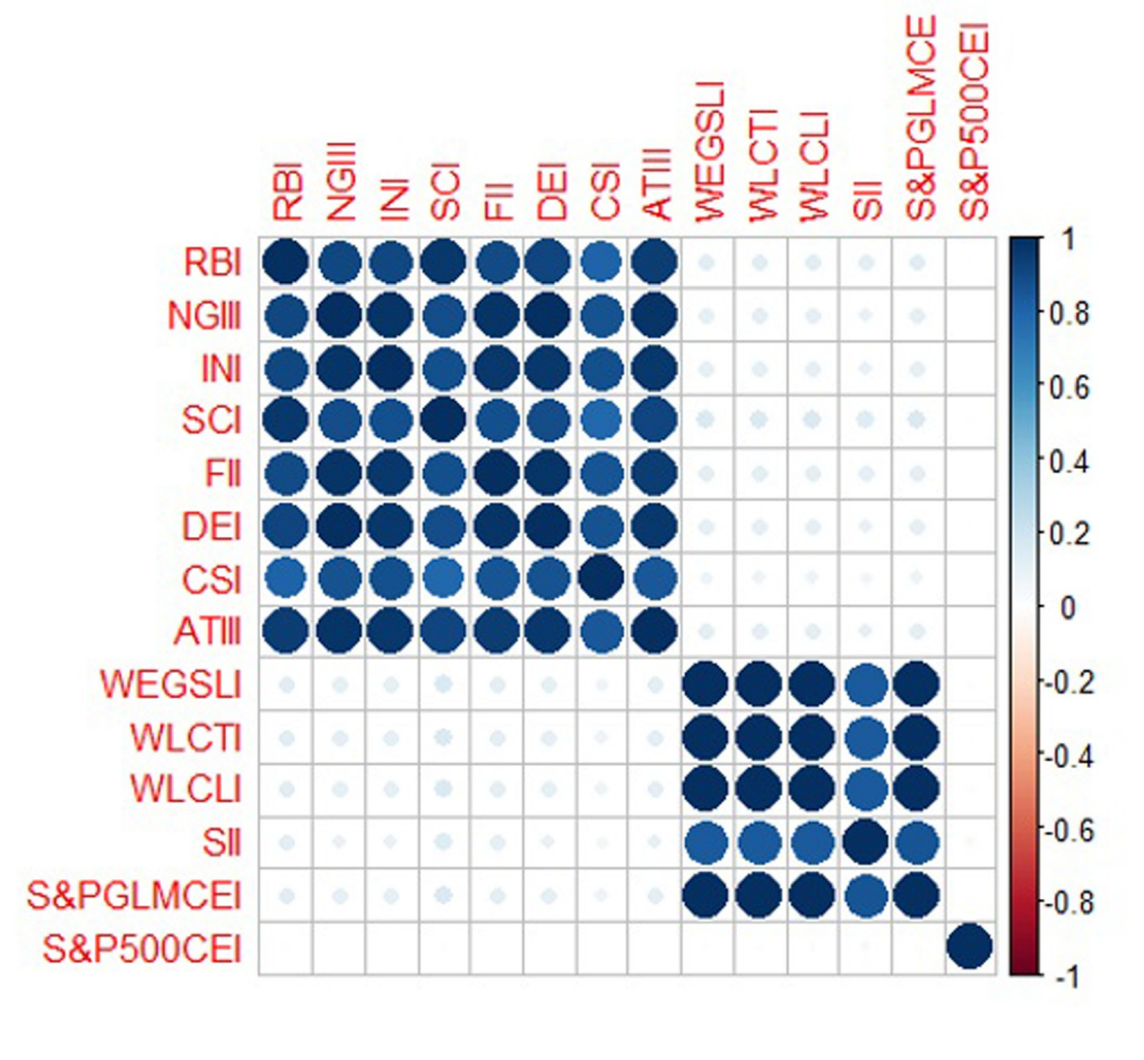
Unconditional correlation matrix.

Fig 3 and Table 2 display the unconditional correlation matrix for the daily return series for thematic and carbon efficient or low carbon indices. Those unconditional correlations were positive and weak except for the correlations with the S&P 500 CEI, which is generally weakly negative across the majority of price returns. Considering the unconditional correlations in the carbon efficient and low carbon indices, the coefficients ranged from 0.1599 for the WLCTI-SCI pair to -0.0165 for the S&P 500 CEI-SCI pair. The correlation among returns in the thematic indices was highly positive, with the same pattern occurred among low carbon or carbon efficient indices. Weak and negative unconditional correlations support the idea of possible diversification benefits, and further investigation is needed to explore the hedging benefits among thematic and low carbon investments.

**Table 2:** ADCC-GJR-GARCH estimates.

**Panel 1: ATIII and Carbon efficient indices**							
*Univariate GJR-GARCH parameters*							
	**ATIII**	**WEGSLI**	**WLCTI**	**WLCLI**	**SII**	**S&PGLMCEI**	**S&P500CEI**
ω_1_(*M*)	0.0007	0.0007	0.0007	0.0007	0.0004	0.0006	0.0007
	*0.0536*	*0.0012*	*0.0032*	*0.0027*	*0.0905*	*0.0093*	*0.0115*
ω_1_(*V*)	0.0688	0.0306	0.0325	0.0314	1.9534	0.0295	0.0469
	*0.0175*	*0.0059*	*0.0059*	*0.0061*	*0.0312*	*0.0058*	*0.0058*
*α*_1_	0.0630	0.0973	0.1004	0.1022	0.0753	0.1020	0.1197
	*0.0970*	*0.0681*	*0.0634*	*0.0560*	*0.0142*	*0.0386*	*0.0621*
*β*_1_	0.8407	0.7897	0.7845	0.7834	0.8597	0.7844	0.7800
	*0.0000*	*0.0000*	*0.0000*	*0.0000*	*0.0000*	*0.0000*	*0.0000*
*d*_1_	0.1365	0.1985	0.2027	0.2058	0.0899	0.2014	0.1743
	*0.0287*	*0.0172*	*0.0120*	*0.0118*	*0.0171*	*0.0108*	*0.0346*
*ADCC parameters*							
*φ*_1_	0.0205						
	*0.0000*						
*θ*_1_	0.9237						
	*0.0000*						
*Θ*_2_	0.0453						
	*0.0000*						
Chi^2^	452.76						
	*0.0000*						
Df.	9.9276						
	*0.0000*						
AIC	-61.8830						
BIC	-61.5982						
**Panel 2: CSI and Carbon efficient indices**							
	**CSI**	**WEGSLI**	**WLCTI**	**WLCLI**	**SII**	**S&PGLMCEI**	**S&P500CEI**
*Univariate GJR-GARCH parameters*							
ω_1_(*M*)	0.0006	0.0007	0.0007	0.0007	0.0004	0.0006	0.0007
	*0.1119*	*0.0012*	*0.0032*	*0.0027*	*0.0905*	*0.0093*	*0.0115*
ω_1_(*V*)	0.0382	0.0306	0.0325	0.0314	1.9534	0.0295	0.0469
	*0.0930*	*0.0059*	*0.0059*	*0.0061*	*0.0312*	*0.0058*	*0.0058*
*α*_1_	0.0371	0.0973	0.1004	0.1022	0.0753**	0.1020	0.1197
	*0.2532*	*0.0681*	*0.0634*	*0.0560*	*0.0142*	*0.0386*	*0.0621*
*β*_1_	0.8985	0.7897	0.7845	0.7834	0.8597	0.7844	0.7800
	*0.0000*	*0.0000*	*0.0000*	*0.0000*	*0.0000*	*0.0000*	*0.0000*
*d*_1_	0.1018	0.1985	0.2027	0.2058	0.0899	0.2014	0.1743
	*0.0017*	*0.0172*	*0.0120*	*0.0118*	*0.0171*	*0.0108*	*0.0346*
*ADCC parameters*							
*φ*_1_	0.0201						
	*0.0000*						
*θ*_1_	0.9211						
	*0.0000*						
*Θ*_2_	0.0423						
	*0.0002*						
Chi^2^	420.77						
	*0.0000*						
Df.	9.8730						
	*0.0000*						
AIC	-61.7677						
BIC	-61.4829						
**Panel 3: DEI and Carbon efficient indices**							
	**DEI**	**WEGSLI**	**WLCTI**	**WLCLI**	**SII**	**S&PGLMCEI**	**S&P500CEI**
*Univariate GJR-GARCH parameters*							
ω_1_(*W*)	0.0007	0.0007	0.0007	0.0007	0.0004	0.0006	0.0007
	*0.0490*	*0.0012*	*0.0032*	*0.0027*	*0.0905*	*0.0093*	*0.0115*
ω_1_(*V*)	0.0576	0.0306	0.0325	0.0314	1.9534	0.0295	0.0469
	*0.0022*	*0.0059*	*0.0059*	*0.0061*	*0.0312*	*0.0058*	*0.0058*
*α*_1_	0.0842	0.0973	0.1004	0.1022	0.0753	0.1020	0.1197
	*0.0569*	*0.0681*	*0.0634*	*0.0560*	*0.0142*	*0.0386*	*0.0621*
*β*_1_	0.8232	0.7897	0.7845	0.7834	0.8597	0.7844	0.7800
	*0.0000*	*0.0000*	*0.0000*	*0.0000*	*0.0000*	*0.0000*	*0.0000*
*d*_1_	0.1428	0.1985	0.2027	0.2058	0.0899	0.2014	0.1743
	*0.0072*	*0.0172*	*0.0120*	*0.0118*	*0.0171*	*0.0108*	*0.0346*
*ADCC parameters*							
*φ*_1_	0.0230						
	*0.0000*						
*θ*_1_	0.9167						
	*0.0000*						
*Θ*_2_	0.0431						
	*0.0002*						
Chi^2^	409.68						
	*0.0000*						
Df.	10.2207						
	*0.0000*						
AIC	-61.9528						
BIC	-61.6680						
**Panel 4: FII and Carbon efficient indices**							
*Univariate GJR-GARCH parameters*							
	**FII**	**WEGSLI**	**WLCTI**	**WLCLI**	**SII**	**S&PGLMCEI**	**S&P500CEI**
ω_1_(*M*)	0.0006	0.0007	0.0007	0.0007	0.0004	0.0006	0.0007
	*0.1400*	*0.0012*	*0.0032*	*0.0027*	*0.0905*	*0.0093*	*0.0115*
ω_1_(*V*)	0.0736	0.0306	0.0325	0.0314	1.9534	0.0295	0.0469
	*0.0055*	*0.0059*	*0.0059*	*0.0061*	*0.0312*	*0.0058*	*0.0058*
*α*_1_	0.0687	0.0973	0.10048	0.1022	0.0753	0.1020	0.1197
	*0.0807*	*0.0681*	*0.0634*	*0.0560*	*0.0142*	*0.0386*	*0.0621*
*β*_1_	0.8285	0.7897	0.7845	0.7834	0.8597	0.7844	0.7800
	*0.0000*	*0.0000*	*0.0000*	*0.0000*	*0.0000*	*0.0000*	*0.0000*
*d*_1_	0.1534	0.1985	0.2027	0.2058	0.0899	0.2014	0.1743
	*0.0038*	*0.0172*	*0.0120*	*0.0118*	*0.0171*	*0.0108*	*0.0346*
*ADCC parameters*							
*φ*_1_	0.0224						
	*0.0000*						
*θ*_1_	0.9202						
	*0.0000*						
*θ*_2_	0.0414						
	*0.0002*						
Chi^2^	408.19						
	*0.0000*						
Df.	10.3657						
	*0.0000*						
AIC	-61.7761						
BIC	-61.4913						

**Panel 5: INI and Carbon efficient indices**							
*Univariate GJR-GARCH parameters*							
	**INI**	**WEGSLI**	**WLCTI**	**WLCLI**	**SII**	**S&PGLMCEI**	**the S&P 500 CEI**
ω_1_(*M*)	0.0005	0.0007	0.0007	0.0007	0.0004	0.0006	0.0007
	*0.1749*	*0.0012*	*0.0032*	*0.0027*	*0.0905*	*0.0093*	*0.0115*
ω_1_(*V*)	0.07122	0.0306	0.0325	0.0314	1.9534	0.0295	0.0469
	*0.0064*	*0.0059*	*0.0059*	*0.0061*	*0.0312*	*0.0058*	*0.0058*
*α*_1_	0.0386	0.0973	0.1004	0.1022	0.0753	0.1020	0.1197
	*0.1747*	*0.0681*	*0.0634*	*0.0560*	*0.0142*	*0.0386*	*0.0621*
*β*_1_	0.8399	0.7897	0.7845	0.7834	0.8597	0.7844	0.7800
	*0*	*0.0000*	*0.0000*	*0.0000*	*0.0000*	*0.0000*	*0.0000*
*d*_1_	0.1852	0.1985	0.2027	0.2058	0.0899	0.2014	0.1743
	*0.0005*	*0.0172*	*0.0120*	*0.0118*	*0.0171*	*0.0108*	*0.0346*
*ADCC parameters*							
*φ*_1_	0.0222						
	*0.0000*						
*θ*_1_	0.9219						
	*0.0000*						
*θ*_2_	0.0418						
	*0.0002*						
Chi^2^	432.05						
	*0.0000*						
Df.	10.2417						
	*0.0000*						
AIC	-61.8418						
BIC	-61.5571						
**Panel 6: NGIII and Carbon efficient indices**							
*Univariate GJR-GARCH parameters*							
	**NGIII**	**WEGSLI**	**WLCTI**	**WLCLI**	**SII**	**S&PGLMCEI**	**the S&P 500 CEI**
ω_1_(*M*)	0.0006	0.0007	0.0007	0.0007	0.0004	0.0006	0.0007
	*0.1075*	*0.0012*	*0.0032*	*0.0027*	*0.0905*	*0.0093*	*0.0115*
ω_1_(*V*)	0.0685	0.0306	0.0325	0.0314	1.9534	0.0295	0.0469
	*0.0033*	*0.0059*	*0.0059*	*0.0061*	*0.0312*	*0.0058*	*0.0058*
*α*_1_	0.0770	0.0973	0.1004	0.1022	0.0753	0.1020	0.1197
	*0.0820*	*0.0681*	*0.0634*	*0.0560*	*0.0142*	*0.0386*	*0.0621*
*β*_1_	0.8291	0.7897	0.7845	0.7834	0.8597	0.7844	0.7800
	*0.0000*	*0.0000*	*0.0000*	*0.0000*	*0.0000*	*0.0000*	*0.0000*
*d*_1_	0.1442	0.1985	0.2027	0.2058	0.0899	0.2014	0.1743
	*0.0099*	*0.0172*	*0.0120*	*0.0118*	*0.0171*	*0.0108*	*0.0346*
*ADCC parameters*							
*φ*_1_	0.0218						
	*0.0000*						
*θ*_1_	0.9202						
	*0.0000*						
*θ*_1_	0.0435						
	*0.0000*						
Chi^2^	427.15						
	*0.0000*						
Df.	10.0862						
	*0.0000*						
AIC	-61.7809						
BIC	-61.4962						

**Panel 7: RBI and Carbon efficient indices**							
*Univariate GJR-GARCH parameters*							
	**RBI**	**WEGSLI**	**WLCTI**	**WLCLI**	**SII**	**S&PGLMCEI**	**S&P 500 CEI**
ω_1_(*M*)	0.0009	0.0007	0.0007	0.0007	0.0004	0.0006	0.0007
	*0.0119*	*0.0012*	*0.0032*	*0.0027*	*0.0905*	*0.0093*	*0.0115*
ω_1_(*V*)	0.0404	0.0306	0.0325	0.0314	1.9534	0.0295	0.0469
	*0.1619*	*0.0059*	*0.0059*	*0.0061*	*0.0312*	*0.0058*	*0.0058*
*α*_1_	0.0354	0.0973	0.1004	0.1022	0.0753	0.1020	0.1197
	*0.3466*	*0.0681*	*0.0634*	*0.0560*	*0.0142*	*0.0386*	*0.0621*
*β*_1_	0.8744	0.7897	0.7845	0.7834	0.8597	0.7844	0.7800
	*0.0000*	*0.0000*	*0.0000*	*0.0000*	*0.0000*	*0.0000*	*0.0000*
*d*_1_	0.1300	0.1985	0.2027	0.2058	0.0899	0.2014	0.1743
	*0.0227*	*0.0172*	*0.0120*	*0.0118*	*0.0171*	*0.0108*	*0.0346*
*ADCC parameters*							
*φ*_1_	0.0202						
	*0.0000*						
*θ*_1_	0.9240						
	*0.0000*						
*θ*_2_	0.0442						
	*0.0001*						
Chi^2^	433.93						
	*0.0000*						
Df.	10.0445						
	*0.0000*						
AIC	-62.2041						
BIC	-61.9193						
**Panel 8: SCI and Carbon efficient indices**							
*Univariate GJR-GARCH parameters*							
	**SCI**	**WEGSLI**	**WLCTI**	**WLCLI**	**SII**	**S&PGLMCEI**	**the S&P 500 CEI**
ω_1_(*W*)	0.0008	0.0007	0.0007	0.0007	0.0004	0.0006	0.0007
	*0.0013*	*0.0012*	*0.0032*	*0.0027*	*0.0905*	*0.0093*	*0.0115*
ω_1_(*V*)	0.0304	0.0306	0.0325	0.0314	1.9534	0.0295	0.0469
	*0.0116*	*0.0059*	*0.0059*	*0.0061*	*0.0312*	*0.0058*	*0.0058*
*α*_1_	0.0584	0.0973	0.1004	0.1022	0.0753	0.1020	0.1197
	*0.0778*	*0.0681*	*0.0634*	*0.0560*	*0.0142*	*0.0386*	*0.0621*
*β*_1_	0.8356	0.7897	0.7845	0.7834	0.8597	0.7844	0.7800
	*0.0000*	*0.0000*	*0.0000*	*0.0000*	*0.0000*	*0.0000*	*0.0000*
*d*_1_	0.1669	0.1985	0.2027	0.2058	0.0899	0.2014	0.1743
	*0.0000*	*0.0172*	*0.0120*	*0.0118*	*0.0171*	*0.0108*	*0.0346*
*ADCC parameters*							
*φ*_1_	0.0214						
	*0.0000*						
*θ*_1_	0.9211						
	*0.0000*						
*θ*_2_	0.0430						
	*0.0002*						
Chi^2^	409.46						
	*0.0000*						
Df.	10.3975						
	*0.0000*						
AIC	-62.6028						
BIC	-62.3181						

## 4. Methods

### 4.1. ADCC-GJR-GARCH model

Previous multivariate GARCH models have enormous limitations and make many assumptions. For instance, the constant conditional correlation (CCC) GARCH model contains an assumption that the correlation will remain constant over time, which is an unrealistic assumption in the case of financial markets. Precedingly, Engle’s [2002] DCC-GARCH model considered conditional correlation as time-variant and introduced a more realistic econometric model. In previous studies, models of a similar nature as the multivariate GARCH model were introduced, such as the GO-GARC and EGARCH. Notably, these multivariate GARCH models could capture constant or dynamic correlations. However, the asymmetric impact of information or volatility clustering is ignored in these models.

As earlier indicated, we consider asymmetric dynamic conditional correlation through the Glosten-Jagannathan-Runkle–generalized autoregressive conditional heteroscedasticity (ADCC-GJR-GARCH) model of Cappiello, Engle [54] and Glosten, Jagannathan [55] to examine the possible hedging role of carbon-efficient assets across thematic indices. The ADCC-GARCH model of Cappiello, Engle [54] builds upon the Engle [56] standard dynamic conditional correlation (DCC-GARCH) model. We used the ADCC-GJR-GARCH model in this study due to its greater relevance and empirical significance. The choice of the ADCC-GJR-GARCH model provides greater relevance to the objectives of our study, as this study aims to explore hedging and safe-haven opportunities by estimating asymmetric dynamic correlations between thematic and carbon efficient indices. In terms of empirical significance, it is understood that bad news or negative shocks predict volatility with greater accuracy in financial markets than positive shocks or good news. In financial time series data, estimating the correlation of an asymmetric nature requires the application of the ADCC-GJR-GARCH (1, 1) model, which can be used to capture volatility clustering or to differentiate the impact of positive and negative news that is overlooked in the simple GARCH (1, 1) model [57]. Thus, our study employed the ADCC-GJR-GARCH model to uncover asymmetric conditional correlations over time.

Univariate GARCH models are fitted to the asset returns in the initial stage. Estimating the asymmetric conditional correlation for both positive and negative standardized returns occur in the second stage. The ADCC-GJR-GARCH model is applied in this study, based on a vector of asset returns where *r*_*t*_ is distributed normally with a mean equal to zero, as follows:

$$r_{t}|I_{t-1}∼N\left(0,H_{t}\right)$$ (1)

$$H_{t}=D_{t}R_{t}D_{t}$$ (2)

$$\varepsilon_{t}=H_{t}^{1/2}{\text{z}}_{t}$$ (3)

$$R=\left[diag{\left(Q_{t}\right)}^{-1/2}\right]Q_{t}\left[diag{\left(Q_{t}\right)}^{-1/2}\right]$$ (4)

where the time-varying conditional correlation matrix is denoted by *R*_*t*_.*H*_*t*_ is a representation of the conditional covariance matrix of the *r*_*t*_,*r*_*t*_ = [*r*_1*t*_*r*_2*t*_]′ vector of returns, which is itself a 2x1 vector of returns and includes returns on any thematic index (*r*_1*t*_) and the corresponding carbon efficient or low carbon index (*r*_2*t*_). The conditional standard deviations from the univariate GARCH models are represented by the diagonal matrix *D*_*t*_. A 2×1 i.i.d. vector of the standardized residuals is represented by *z*_*t*_, with the conditional correlation matrix of the standardized residuals being indicated by *Q*_*t*_. Finally, ε_*t*_ = [ε_1*t*_,ε_2*t*_]^′^ is a 2×1 vector of conditional residuals on the dataset at time *t*-1.

The asymmetric univariate GJR-GARCH (1,1) model of Cappiello, Engle [54] was used to estimate the component of *H*_*t*_ in line with the work of Glosten, Jagannathan [55] as follows:

$$h_{i,t}=\omega_{i}+\alpha_{i}\varepsilon_{i,t-1}^{2}+\beta_{i}h_{i,t}+d_{i}\varepsilon_{i,t-1}^{2}I\left(\varepsilon_{i,t-1}\right)$$ (5)

where the conditional variance of the return series is represented by *h*_*i*,*t*_, and the asymmetric or GJR term is noted as *d*_*i*_. Additionally, the persistence of the volatility process (GARCH) is assessed by *β*_*i*_, the ARCH effect is denoted by *α*_*i*_, and *ω*_*i*_ denotes a constant term. If *ε_i,__t_*_-1_ < 0, then the indicator function *I*(ε_*i*,*t*-1_) = 1 and if these conditions are satisfied, it is equal to zero. If the assumption or condition of *α*_*i*_ *+ β*_*i*_ + *d*_*i*_ < 1 and *α*_*i*_ > 0 are satisfied, then steadiness and positivity are both confirmed.

The standardized residuals *z*_*t*_ are used to calculate the conditional correlation parameters after estimation by the univariate GARCH models. Based on the ADCC-GARCH model, *Q*_*s*_ dynamics are described as follows:

$$Q_{t}=\left(1-\theta_{1}-\theta_{2}\right)Q-\varphi N+\theta_{1}\left({\text{z}}_{t-1}{\text{z}}_{t-1}^{'}\right)+\theta_{2}Q_{t-1}+\varphi \left(\eta_{t-1}\eta_{t-1}^{\prime }\right)$$ (6)

The unconditional correlation matrix of *η*_*t*_ is written as $N_{j}=E[\eta_{t},\eta_{t}^{'}]$, while *z*_*t*_ is written as $Q_{j}=[Ez_{t},z_{t}^{'}]$]. When the parameter is true, the indicator function is given by $Q_{j}=[Ez_{t},z_{t}^{'}]$. The function *η*_*t*_ = *I*(*zt*,<0) ∘ *z_t_* assumes that if the coefficient is equal to zero, the argument is false and that, if it is one, the argument is true. The parameter matrices are represented by *θ*_1_,*θ*_2_ and *φ*, while “∘” denotes the Hadamard product. If *φ* = 0, the AGDCC-GARCH model is reduced to the conventional DCC-GARCH model without an unequal influence on the conditional correlation.

The time-varying correlation matrix for the ADCC-GJR-GARCH model is shown as follows:

$$R_{t}=Q_{t}^{*}Q_{t}Q_{t}^{*-1}$$ (7)

where the diagonal matrix is represented by $Q_{t}^{*}$, and $Q_{t}^{*}$ has the square root of the *ith* diagonal of *Q*_*t*_ in the *ith* diagonal position.

### 4.2. Wavelet coherence approach

Empirical analysis was also conducted in this study using bivariate wavelet coherence analysis. Wavelet coherence is among the most widely recognized techniques for measuring the co-movements among financial and economic time series. In contrast to standard time series modeling, the underlying approach enables the estimation of the co-movements between any two time series across both time and frequency realms [58].

Following the wavelet coherency method of Torrence and Compo [59], the cross wavelet transform between two time series, x(t) and y(t), is defined as follows:

$$W_{x,y}(a,b)=W_{x}{\left(a,b\right)}^{*}W_{y}(a,b)$$ (8)

where W_x_ (*a*,*b*) and W_y_ (*a*,*b*) are the cross wavelet transforms of x(t) and y(t), respectively, and ’a’ and ’b’ are the scale and position index, respectively. Furthermore, * denotes a complex conjugate. The cross-wavelet transform can capture the local covariance between two time series, x(t) and y(t), at each scale.

After Torrence and Compo [59], we followed Goodell and Goutte [58] to define the wavelet coherence between any two time series as

$$R^{2}(u,s)=\frac{{\left|S\left[b^{-1}W_{i,j}(a,b)\right]\right|}^{2}}{S\left[b^{-1}{\left|W_{a}(a,b)\right|}^{2}\right]S\left[b^{-1}{\left|W_{j}(a,b)\right|}^{2}\right]},\;\text{with}\;R^{2}(a,b)\in [0,1]$$ (9)

The squared wavelet coherence between x(t) and y(t) identifies notable co-movement through cross-wavelet power series at each scale, where *R*^2^(*a*,*b*) describes the localized correlation in a time-frequency domain in squared form, and its value falls between 0 and 1. Moreover, *S* denotes a smoothing operator for each scale 0 ≤ R^2^ (*u*,*s*) ≤ 1. Since *R*^2^(*a*,*b*) is limited to only positive values, the wavelet coherence phase difference is introduced at this step of the methodology to reflect both positive and negative correlations. Thus, the wavelet coherence phase difference can be defined as:

$$\rho_{i,j}(a,b)=tan^{-1}\left(\frac{lm\left\{S\left(b^{-1}W^{i,j}(a,b)\right)\right\}}{Re\left\{S\left(b^{-1}W^{i,j}(a,b)\right)\right\}}\right),\text{with}\;\rho_{ij}\in [-\pi ,\pi ]$$ (10)

where *lm* denotes the imaginary smoothed part, and *Re* represents the real part of the smoothed cross-wavelet transform.

## 5. Empirical results and discussions

### 5.1. ADCC-GJR-GARCH estimates

The Akaike Information Criterion (AIC) and Bayesian Information Criterion (BIC) lag orders were used to select the best marginal model, i.e., those that maximize log-likelihood while minimizing AIC and BIC [57]. Table 2 reports the estimates for the ADCC-GJR-GARCH model. The parameters of the estimated model are statistically significant at the significance levels of 1% and 5%. The autoregression of the order-one coefficient is significant, except for ATIII and RBI, showing that the current return estimates are unaffected by the one-day price returns of ATIII and RBI. Considering the significance of the parameters for the majority of indices, this indicates that past price returns influence current returns, indicating possible market inefficiency. Moreover, there was evidence of statistically significant persistence in both short- and long-term volatility, as shown by coefficients *α*_*i*_ and *β*_*i*_, respectively, suggesting that thematic and carbon efficient indices exhibit volatility clustering. Notably, long-term volatility persistence was greater than short-term volatility persistence, and the conditional volatility of indices tends to increase quickly because past conditional volatility has a greater impact than return innovations. These findings can give portfolio managers insights into the application of trading techniques based on long-run volatility persistence due to their potential explanatory power regarding present volatility.

Additionally, the parameter (*d*_*i*_) indicates the asymmetric coefficient in the ADCC model, which is statistically significant for all returns series. The evidence for the significance of *d*_*i*_ demonstrates the influence of negative shocks (i.e., bad news) on conditional volatility dynamics. The leverage effect for all return series is positive, indicating that negative shocks tend to increase the conditional volatility to a greater degree than positive shocks of comparable magnitudes. The stability of our ADCC model was confirmed, as we noted that the total of the *α*_*i*_ (short-term), *β*_*i*_ (long-term) and *d*_*i*_ (asymmetric) volatility parameters was less than one (< 1). Notably, the impact of bad news (*α*_*i*_ + *d*_*i*_) is greater in magnitude than that of good news (*α*_*i*_). Hence, the positive leverage term parameters for the ADCC model are statistically significant and nonnegative, showing the appropriateness of using the ADCC-GJR-GARCH model. These results reveal that SCI (10.3975) has the highest estimated degree-of-freedom and CSI (9.8730) has the lowest degree-of-freedom (Df.). The degree of freedom or shape parameter of student-*t* distribution random errors is statistically significant and greater than two (> 2), confirming that the leptokurtic behavior of estimated residuals is captured by the degree of freedom (i.e., it is fat-tailed).

Regarding the asymmetric dynamic conditional correlation parameters, the estimates displayed in Table 2 show that the parameters of the ADCC model are statistically significant with positive signs across panels (Panel 1 to 8) and that the sum of ADCC parameters (*α*_*i*_ + *β*_*i*_ + *d*_*i*_) is less than one (< 1). These findings indicate that the asymmetric dynamic conditional correlations between thematic and low carbon emissions were mean reverting. Moreover, the parameter (*α*_*i*_) emphasizes the significance of shocks between the thematic and low carbon price returns, whereas *β*_*i*_ shows stronger persistence-of-volatility between markets, while the third parameter (*d*_*i*_) indicates the asymmetric effect of price returns.

### 5.2. Asymmetric dynamic conditional correlations

The second stage of the ADCC-GJR-GARCH model is performed to obtain the average coefficients of asymmetric conditional correlation. Considering the panel-specific asymmetric conditional correlations, Table 3 demonstrates the asymmetric conditional correlation for each panel from Panel 1 to Panel 8.

**Table 3:** Asymmetric dynamic conditional correlations.

**Panel 1: DCCs between ATII and carbon efficient indices**				
**Pairs**	**Coefficients**	**Std. error**	**t value**	**p value**
ATIII-WEGSLI	-0.0672	0.0514	-1.3070	0.1915
ATIII-WLCTI	-0.0660	0.0516	-1.2790	0.2012
ATIII-WLCLI	-0.0669	0.0515	-1.2970	0.1949
ATIII-SII	-0.0382	0.0501	-0.7638	0.4452
ATIII-S&PGLMCEI	-0.0655	0.0515	-1.2730	0.2033
ATIII-S&P500CEI	-0.1447	0.0548	-2.6400	0.0084
**Panel 2: ADCCs between CSI and Carbon efficient indices**				
**Pairs**	**Coefficients**	**Std. error**	**t value**	**p value**
CSI-WEGSLI	-0.063574	0.051384	-1.237000	0.216300
CSI-WLCTI	-0.061629	0.051765	-1.191000	0.234100
CSI-WLCLI	-0.062174	0.051789	-1.201000	0.230200
CSI-SII	-0.054392	0.049393	-1.101000	0.271100
CSI-S&PGLMCEI	-0.059593	0.051494	-1.157000	0.247400
CSI-S&P500CEI	-0.109416	0.050079	-2.185000	0.029100
**Panel 3: ADCCs between DEI Carbon efficient indices**				
Pairs	**Coefficients**	**std. error**	**t value**	**p value**
DEI-WEGSLI	-0.0622	0.0501	-1.2400	0.2154
DEI-WLCTI	-0.0599	0.0503	-1.1910	0.2340
DEI-WLCLI	-0.0611	0.0502	-1.2150	0.2246
DEI-SII	-0.045	0.0498	-0.9171	0.3593
DEI-S&PGLMCEI	-0.0597	0.0502	-1.1890	0.2348
DEI-S&P500CEI	-0.1242	0.0529	-2.3450	0.0192
**Panel 4: ADCCs between FII and Carbon efficient indices**				
**Pairs**	**Coefficients**	**std. error**	**t value**	**p value**
FII-WEGSLI	-0.0591	0.0505	-1.1710	0.2417
FII-WLCTI	-0.0545	0.0506	-1.0770	0.2818
FII-WLCLI	-0.0558	0.0506	-1.1030	0.2703
FII-SII	-0.0358	0.0502	-0.7135	0.4757
FII-S&PGLMCEI	-0.0551	0.0507	-1.0870	0.2775
FII-S&P500CEI	-0.1184	0.0522	-2.2700	0.0234
**Panel 5: ADCCs between INI and Carbon efficient indices**				
Pairs	**Coefficients**	**std. error**	**t value**	**p value**
INI-WEGSLI	-0.0697	0.0510	-1.3650	0.1726
INI-WLCTI	-0.0657	0.0512	-1.2840	0.1995
INI-WLCLI	-0.0668	0.0512	-1.3060	0.1930
INI-SII	-0.0531	0.0505	-1.0520	0.2932
INI-S&PGLMCEI	-0.0640	0.0511	-1.2530	0.2106
INI-S&P500CEI	-0.1449	0.0545	-2.6570	0.0080

**Panel 6: ADCCs between NGIII and Carbon efficient indices**				
**Pairs**	**Coefficients**	**std. error**	**t value**	**p value**
NGIII-WEGSLI	-0.0654	0.0503	-1.3000	0.1940
NGIII-WLCTI	-0.0632	0.0504	-1.2530	0.2103
NGIII-WLCLI	-0.0647	0.0504	-1.2850	0.1991
NGIII-SII	-0.0466	0.0504	-0.9244	0.3555
NGIII-S&PGLMCEI	-0.0634	0.0504	-1.2590	0.2085
NGIII-S&P500CEI	-0.1327	0.0538	-2.4670	0.0138
**Panel 7: ADCCs between RBI and Carbon efficient indices**				
**Pairs**	**Coefficients**	**std. error**	**t value**	**p value**
RBI-WEGSLI	-0.0597	0.0502	-1.1880	0.2351
RBI-WLCTI	-0.0593	0.0507	-1.1700	0.2423
RBI-WLCLI	-0.0595	0.0506	-1.1760	0.2397
RBI-SII	-0.0328	0.0488	-0.6729	0.5012
RBI-S&PGLMCEI	-0.0583	0.0505	-1.1550	0.2485
RBI-S&P500CEI	-0.1774	0.0547	-3.2410	0.0012
**Panel 8: ADCCs between SCI and Carbon efficient indices**				
**Pairs**	**Coefficients**	**std. error**	**t value**	**p value**
SCI-WEGSLI	-0.0697	0.0510	-1.3650	0.1726
SCI-WLCTI	-0.0657	0.0512	-1.2840	0.1995
SCI-WLCLI	-0.0668	0.0512	-1.3060	0.1930
SCI-SII	-0.0531	0.0505	-1.0520	0.2932
SCI-S&PGLMCEI	-0.0640	0.0511	-1.2530	0.2106
SCI-S&P500CEI	-0.1449	0.0545	-2.6570	0.0080

The outcome of Panel 1 illustrates the ADCCs of the Autonomous Technology & Industrial Innovation Index with major low carbon and sustainable indices (S&P and MSCI). The correlation coefficients among these pairs (ATIII-WEGSLI, ATIII-WLCTI, ATIII-WLCLI, ATIII-SII and ATIII-S&PGLMCEI) were negative and statistically nonsignificant. However, the ADCC coefficient sign was negative and statistically significant for the ATIII-S&P500CEI pair, suggesting an inverse relation between that pair. Similarly, the remaining panels (Panel 2 ∼ Panel 8) show asymmetric relationships among the returns of thematic and low carbon emission indices. These findings indicate that the returns of thematic or transformative indices (the Autonomous Technology & Industrial Innovation, Cybersecurity, Digital Economy, Fintech Innovation, Innovation, Next Generation internet Innovation, and Robotics and Smart Cities Indices) are uncorrelated with the carbon efficient indices (the MSCI World Low Carbon Leaders, MSCI World Low Carbon Target, S&P Global LargeMidCap Carbon Efficient, MSCI ACWI Sustainable Impact and World EGS Leader Indices. In contrast, the transformative indices reveal a negative asymmetric relationship with the S&P 500 Carbon Efficient Index across panels (Panel 2 ∼ Panel 8). In our case, the negative trajectory of ADCCs also validates the weak hedge (safe-haven) properties during both tranquil and tumultuous periods (represented by the global health crisis).

Overall, Table 3 illustrates the results of asymmetric nature conditional correlation for Panels 1∼8, which are time variant. Regarding the sign of the correlation coefficients, the average coefficients were predominantly negative across all panels (1∼8). Of equal importance, the correlation coefficients of thematic indices with returns of carbon efficient indices were statistically nonsignificant aside from the S&P 500 carbon efficient index (S&P500CEI), as shown in Panels 1∼8. Based on the strong (weak) hedge or safe-haven definitions of Baur and Lucey [60], Bouri, Jalkh [61], Bouri, Molnár [62], Gustafsson, Dutta [63], assets that are uncorrelated (negatively correlated) can on average serve as a weak (strong) hedge instrument. In our case, the nonsignificant correlation suggests that the majority of thematic indices are uncorrelated with carbon efficient indices, which validates the role of carbon efficient indices as weak hedges for thematic indices. Among all carbon efficient indices, the S&P 500 CEI shows negative significant correlation coefficients with the thematic indices across Panels 1∼8; hence, the S&P 500 carbon efficient index possesses rich properties of a strong hedge (safe-haven). These findings corroborate the results of Apostolou and Papaioannou [51], who emphasized that low carbon indices such as the S&P 500 CEI could present hedging or diversification opportunities for investors aiming to rebalance their portfolios away from brown assets due to the high risk associated with brown investments.

Similarly, employing a rolling window multiple correlation technique, Fareed, Abbas [29] reported a positive relationship between the S&P 500 carbon efficient index and the COVID-19 pandemic, suggesting safe-haven properties of S&PCEI during this period. Koçak, Bulut [34] also reported a positive impact of COVID-19 on the S&P 500 CEI during the global health crisis. The strong hedge or safe-haven can also be supported by the fact that the S&P 500 Carbon Efficient Index weights companies based on carbon intensity, while the Carbon Efficient Select Index weights companies based on their overall carbon footprint [13]. The S&P 500 carbon efficient index gains considerably in the recent turmoil period (global health crisis), as evidenced by earlier research [34] supporting the safe-haven role of S&PCEI. In general, financial investors trust low-carbon companies more than companies or financial assets in other sectors, resulting in investing in such company shares even during a health crisis. Our findings are consistent with earlier studies that reported the S&P 500 carbon efficient index as a hedging or safe-haven tool and investment opportunity for mitigating the economic fragility (global health crisis) and risks of thematic investments.

**Fig 4:**
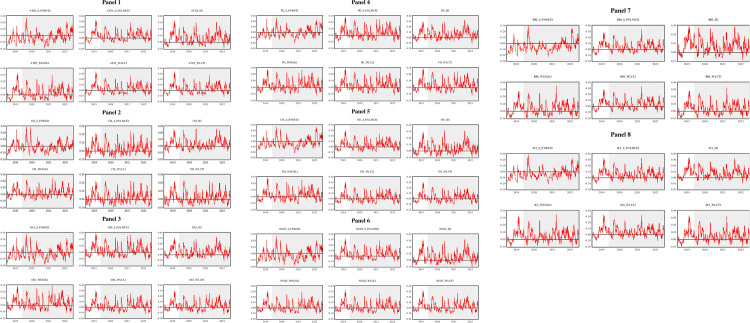
Asymmetric DCCs among the thematic and carbon efficient indices.

The asymmetric conditional correlation coefficients for thematic and carbon efficient indices using the GJR-GARCH model are depicted in Fig 4. Overall, as indicated in subfigures (a∼h), we see inconsistent negative and larger negligible positive correlation jumps among all thematic and carbon efficient indices. The heterogeneous negligible positive/negative trajectory of the correlation ranging from January 1^st^ 2019 to November 30^th^ 2019 refers to the pre-COVID or tranquil period, where low carbon indices show weak hedges or diversifier characteristics with thematic indices (see subfigures a∼h), aside from the S&P 500 carbon efficient index, which serves as a strong hedge against thematic indices. Notably, the asymmetric correlation graphs also exhibited time-variant and heterogeneous behavior during COVID-19. Negative shifts were not intensive and stable, referring to the global health crisis where nonsignificant negative correlations (uncorrelated) confirm the claim that carbon efficient indices acted as weak safe-havens during the turmoil period. However, the magnitude of the negative shocks, except for the S&P 500 carbon efficient index, was not intensive during the turmoil period of COVID-19 (see subfigures a∼h). All thematic indices revealed a negative significant asymmetric correlation with the S&P 500 carbon efficient index throughout the sample period, validating that the S&P 500 carbon efficient index acts as the most consistent strong hedge and safe-haven for thematic indices during both tranquil and tumultuous periods. When deciding on future portfolios, sustainable investors need to be more willing to invest in green enterprises when combined with long-term sustainable development goals, which would advance carbon efficiency and decarbonization [8,43,64]. These findings are a result of the quick rise in COVID-19 transmission and the widespread implementation of lockdown measures that occurred at the beginning of the pandemic. Later, lockdown resulted in the closure of almost every sector worldwide, including industrial and service sectors, which is the main reason for S&PCEI stock returns to have experienced a significant fluctuation and negative correlation during the global health crisis. Moreover, the increased use of digital and innovative technologies during lockdown fostered social, financial, and economic transformation. However, the majority of thematic indices failed to exert carbon efficiency, although these thematic indices are innovative, highly technology focused and reach every part of human lives. In summary, this implies that the S&PCEI and other low carbon indices are strong (weak) hedges or safe-havens for thematic investors in both tranquil and tumultuous periods (COVID-19 pandemic). Thematic investors should diversify their risk by building a portfolio and preferably investing in S&PCEI rather than in other carbon efficient indices due to their weak hedging or safe-haven power. These results are consistent with those of earlier research [9,27], which recommends pursuing socially responsible assets to earn social and financial rewards.

### 5.3. Wavelet coherence evidence

**Fig 5:**
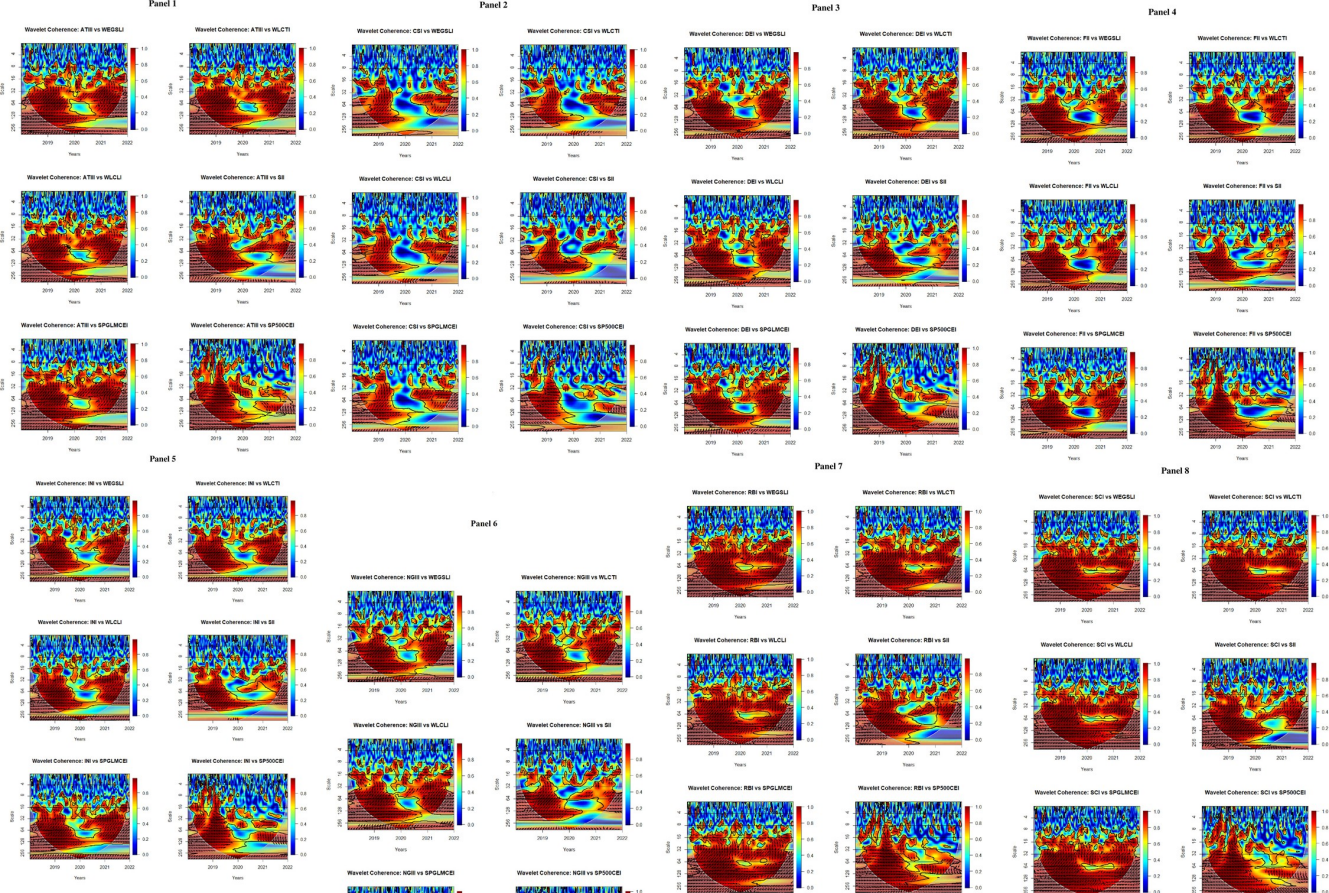
Wavelet coherence among thematic and carbon efficient indices.

This section presents the results of wavelet coherence analysis between thematic and carbon efficiency indices. Fig 5 represents the co-movement across both time and frequency. In addition, it also reveals the lead-lag-centered phase difference. Each subfigure displayed in Fig 5 has five key outputs. First, the horizontal axes (vertical axes) represent the time (frequency) domain. The component of frequency is decomposed into seven scales, where the shortest scale (2–4 days) represents the highest frequency band, and the longest level (128–256 days) denotes the lowest frequency band. Furthermore, we classified these frequency scales into three timeframes relative to investment horizons, i.e., short-term horizon (2–4, 4–8, 8–16, 16–32 days), medium-term horizon (32–64 and 64–128 days) and long-term horizon (128–256 days). Second, the magnitude of the co-movement is indicated by a color band where warmer colors (red and yellow) indicate higher co-movement or interlinkages, whereas colder colors (blue and green) highlight lower co-movement or coherence. Third, the black contour represents significance at the 0.05 or 5% level as calculated with the Monte Carlo simulation technique. Fourth, the curved dense line indicates the cone of influence, which shows the area impacted by edge effects. Additionally, the area beyond the cone of influence represents nonsignificant interlinkages. Finally, the directed arrows toward eight different directions (←,→,↑,↓,↘,↗,↙,↖) signify the lead-lag-based phase difference and co-movement directions (positive or negative where zero phase difference indicates a precisely aligned co-movement of series; arrows pointing to the right (left) side → (←) represents that time series are in-phase (out-phase). The in-phase (out-phase) status depicted by the arrows denotes positive (negative) co-movement between thematic and carbon efficient indices. Arrows directing to the right-up or left-down (↗ and ↙) denote that innovation-themed indices lead the outcomes over carbon efficient indices. In contrast, right-down or left-up arrows (↘ and ↖) suggest that carbon-efficient indices lead the outcomes over innovation-themed indices. To conclude, straight up or straight down arrows (↑,↓) indicate that carbon efficient indices are leading or lagging, respectively.

Following these key chunks of wavelet coherence graphs, we discuss the wavelet coherence analysis between thematic and carbon efficient indices across short-term, medium-term, and long-term investment horizons. In the case of Fig 5 (Panel 1 ∼ Panel 8), we observe low coherence dominating the entire sample period, as Fig 5 (Panel 1 ∼ Panel 8) appears to be mostly blue across the 2-4- and 4-8-day scales. However, it can be noted that the power spectrum shows high coherence (in red) at the 8-16- and 16-32-day scales with left-directed arrows (←). These results suggest that the underlying markets are out-phase in the short term, which indicates weak and negative co-movement in the short-term horizon. The out-phase or negative co-movements suggest short-lived weak (2–4, 4–8 days) and strong (8–16 and 16–32 days) hedging or safe-haven opportunities. In addition, right-down or left-up arrows (↘ and ↖) in the short run suggest that carbon efficient indices are leading the thematic indices in the short-term investment horizon.

In contrast, we noted high coherence power in red across the 32-64- and 64-128-day scales (Panel 1 Panel 8), and the majority of the arrows are right-directed (→). The black line contours are more noticeable on 32-64- and 64-128-day scales, and they are significant at the 5% level. These results also specify that underlying indices are in-phase in the medium-term investment horizon, which indicates strong positive co-movement across the subsequent investment horizon. In addition, the in-phase or strong positive co-movement implies minimal or no diversification opportunities in underlying asset classes in medium-term and long-term investment horizons during this period. Moreover, right-up or left-down (↗ and ↙) arrows in the underlying frequency scale suggest that thematic indices are leading the outcomes over carbon efficient indices in the medium-term investment horizon.

Similar to the medium-term investment horizon, high coherence can be noted beginning in 2019 to 2020 on a 128–256-day scale as noted in red (Panel 1 ∼ Panel 8). Thick black line contours in red areas during the respective period imply high co-movement significance at the 5% level. From 2019 to the end of 2020, the right directions arrows (→) denote in-phase and positive co-movement of the thematic and carbon efficient indices in the long-term investment horizon (Panel 1 ∼ Panel 8). Moreover, right-up or left-down arrows (↗ and ↙) on the 128–256-day scale suggest that thematic indices lead the outcomes in carbon efficient indices in the long-term investment horizon during the early sample period. Noticeably, a persistent yellow spot can be observed from 2021 to the end of the sample in the long-term horizon for each pair of thematic carbon-efficient indices, which indicates weak coherence during the COVID-19 pandemic. These findings imply minimal diversification properties in 2019 and 2020; however, carbon-efficient investments hold weak safe-haven properties.

Overall, it is determined in this discussion that the co-movements between underlying asset classes are out-phase (in phase) in the short term (medium term and long term), which indicates negative co-movement in the short term and positive co-movement in medium-term and long-term investment horizons. In addition, thematic investments lead (lag) the outcomes in carbon efficient assets in the medium run and long run (short run). Considering the hedging perspective, carbon-efficient investments are hedges or safe-havens for thematic investments in the short run, but they are limited to weak hedges or safe-havens in the long run. These results also corroborate our ADCC-GJR-GARCH findings, as the DCC-GJR-GARCH results show that the asymmetric correlation between thematic and carbon efficient indices is weak or uncorrelated (negative but nonsignificant estimates) and negative (in the case of the S&P 500 Carbon Efficient Index). However, in the case of the wavelet coherence approach, the findings reveal notable positive co-movement between thematic and carbon efficient indices in medium-term and long-term investment horizons. These findings also support the idea of considering innovative technologies [11,18,65] to mitigate CO_2_ and stimulate carbon efficiency. As a notion that CO_2_ and carbon efficiency are inversely correlated across multiple horizons was developed in earlier research [66], the role of thematic investments following the use of digitalization, urbanization, and innovative technology can support the fight against rising carbon emissions to ensure carbon efficiency. In addition, stringent environmental policies and green technological development [67] can be leveraged to pave the way for environmental quality and climate challenges.

## 6. Concluding remarks

Carbon efficiency has become a key global concern. However, whether innovation- and digitalization-based thematic investment can foster carbon emissions efficiency remains a key question in the current digital era. Hence the asymmetric dynamic correlation and coherence between major thematic and carbon efficient indices from January 2019 to January 2023 is examined in this paper through the use of ADCC-GJR-GARCH and wavelet coherence techniques. The co-movements between underlying asset classes following the lead-lag mechanism at different time-frequencies to explore the time-frequency-based hedging or safe-haven opportunities are also examined from a global perspective.

The empirical analysis results of the ADCC-GJR-GARCH highlight some considerable conclusions. The findings reveal a negative and nonsignificant correlation between thematic investing indices and carbon efficient investments, aside from the S&P 500 carbon efficient index, which rather shows a consistent significant negative correlation. The other carbon efficient indices show a nonsignificant negative association over time, which reveals a weak or nonnegligible impact of thematic investment on carbon efficiency. Using the ADCC-GJR-GARCH hedge or safe-haven characteristics of low carbon emissions indices reveals that the S&P 500 carbon efficient index serves as a strong hedge or safe-haven investment for thematic investments, whereas MSCI World Low Carbon Leaders, MSCI World Low Carbon Target, S&P Global LargeMidCap Carbon Efficient, MSCI ACWI Sustainable Impact and World EGS Leader indices are weak hedges or safe-havens assets during periods of tranquility or tumult (COVID-19). The strong hedge or safe-haven feature of the S&P 500 carbon efficient index indicates that adding S&P 500 carbon efficient stocks to a thematic investment portfolio result in considerable hedging or diversification benefits for innovation-themed and responsible investors who tend to bear less risk during economic stress and normal periods. Hence, carbon efficient investments, particularly the S&P 500 carbon efficient index, can absorb economic shocks during the global health crisis or fragile periods and during the normal state of the economy. In addition, their inclusion in the portfolio gives innovation-themed, socially responsible, and sustainable investors the opportunity to include strong/weak hedging or safe-haven assets in portfolios to avert their risks and vulnerabilities.

Considering the volatility consistency results, thematic and carbon efficient investments are considered as inefficient over time and reject market efficiency. These findings can provide portfolio managers with insights into the application of trading techniques based on long-run volatility persistence due to their potential explanatory power on present volatility. Implicatively, this suggests that investors can earn abnormal gains by predicting the future prices of these assets. Thus, innovation-themed, sustainable, and socially responsible investors can become motivated to invest in carbon efficient investments during periods of opportunity for overcoming crises. In addition, these investments also offer greater social and environmental benefits, as the underlying thematic investments are immune to carbon emissions and they promote carbon efficiency during the stress period.

Furthermore, the wavelet coherence analysis noted an identical interconnectivity between thematic and carbon efficient indices across different time-frequency scales. The results of wavelet coherence between underlying asset classes highlight negative weak co-movement in the short term and positive strong co-movement in medium- and long-term investment horizons or frequency scales. In addition, thematic investments lead (lag) the outcomes in carbon efficient assets in the medium- and long-term (short-term) time-frequencies. Considering the hedging perspective, carbon-efficient investments can serve as hedges or safe-havens for thematic investments in the short-term investment horizon but are only weak hedges or safe-havens in the long-term horizon following the weak coherence that occurred from 2021 to 2022. Eventually, the results of wavelet coherence support the findings of the ADCC-GJR-GARCH model.

These findings have insightful implications for socially responsible investors, policymakers, and regulatory bodies across the globe. S&P 500 carbon efficient investment provides a greater opportunity for combatting the intensity of economic shocks on portfolios, as it acts as a strong hedge or safe-haven asset during a global health crisis. The remaining carbon-washing indices, i.e., MSCI World Low Carbon Leaders, MSCI World Low Carbon Target, S&P Global LargeMidCap Carbon Efficient, MSCI ACWI Sustainable Impact and World EGS Leader indices, also offer diversification benefits, as these are weak hedge or safe-haven assets. Nevertheless, comparing the diversification or hedging potential of low carbon assets and S&P 500 carbon efficient index shows a greater capacity to minimize or diversify the economic or financial risk of investment portfolios. Therefore, investors need to focus on dynamic and heterogeneous trends and jumps in the correlation across tranquil and tumultuous economic periods. In addition, investors need to consider short-term investment horizons for optimal financial gains. Moreover, responsible investors should choose hedging or diversification instruments to deal with and avoid uncertain economic conditions.

Policy-makers and regulatory bodies can encourage investors to make carbon-efficient investments and encourage companies and financial institutions to issue carbon-efficient stocks or investments to safeguard social and economic risks during fragile periods. As advocated by Breitenstein, Nguyen [68], financial institutions can lower their risk exposure by demonstrating a strong commitment to environmental responsibility and performance. Equally important, companies that engage in thematic investments should intervene in thematic or transformative technological companies to strengthen the sustainable digital economy and technology transfer processes. A considerable degree of positive interconnectedness also highlights the usefulness of innovation-themed investment to building and improving a carbon-efficient planet. Governments can also focus on these technological innovations and thematic investments by adopting measures to evaluate their operational and financial requirements and to promote green and sustainable investments [9] for achieving carbon efficiency targets. For instance, governments may consider the use of carbon taxes or other taxation policies rather than relying on carbon trading.

Our study has few limitations that can guide future research endeavors. Although the findings of current studies are significant, it is important to highlight further potential research into thematic investing using a diverse range of MSCI thematic indices. This study is focused on those thematic indices that are relative to innovation or technological aspects of global society. Further research needs to be conducted to consider other thematic indices, such as the MSCI disruptive technology index, MSCI future mobility index, MSCI space exploration index or MSCI blockchain economy index. We used a rather small sample covering 1 January 2019 to 30 January 2023 period due to daily data unavailability. While this provides a snapshot of the market during the COVID-19 crisis, the relatively short timeframe may limit the ability to draw robust conclusions or capture longer-term trends and patterns. More research can be conducted using monthly data frequency over a larger sample to generalize our findings and give a clearer picture of the hedge, safe-haven, and diversification characteristics of carbon-efficient investments in long-term. In the future, scholars and academicians can integrate a comprehensive set of thematic and low carbon indices in a portfolio mix to assess hedging and diversification potential. Another potential future direction is to assess the interconnectedness of country-specific thematic and low carbon investing considering the MSCI US Tech 125 Index or MSCI China Tech Indices as possibilities. Finally, a variety of alternative multivariate methodologies could be used to investigate connectedness between the thematic and carbon efficient indices (time-varying parameter-vector autoregressions) to reveal dynamic spillovers and portfolio diversification strategies.

## Supporting information

S1 File (DOCX)

S1 Data (XLSX)
