# Supplementary material for: Asymmetric and time-frequency co-movements among innovation-themed investments and carbon emission efficiency: Thematic investing and hedging opportunities
Source: PLoS One. 2024 Feb 29;19(2):e0293929. doi: 10.1371/journal.pone.0293929 (PMC10903807; doi:10.1371/journal.pone.0293929)
Supplement: S1 File — (DOCX) [file pone.0293929.s001.docx]

**Appendix A:**

**Table A1. Descriptive statistics level series**

| **Variables** | **M** | **Max.** | **Min.** | **SD.** | **Skew.** | **Kurt.** | **JB** | **Obs.** |
| --- | --- | --- | --- | --- | --- | --- | --- | --- |
| ATIII | 2242.407 | 3398.818 | 1299.884 | 596.098 | 0.058 | 1.688 | 75.569* | 1045 |
| CSI | 2314.514 | 3331.606 | 1359.868 | 536.204 | 0.056 | 1.690 | 75.361* | 1045 |
| DEI | 2524.424 | 3606.771 | 1515.388 | 605.561 | 0.148 | 1.636 | 84.896* | 1045 |
| FII | 2455.116 | 3613.661 | 1474.392 | 598.700 | 0.244 | 1.599 | 95.912* | 1045 |
| INI | 2316.522 | 3392.741 | 1408.938 | 578.073 | 0.278 | 1.734 | 83.315* | 1045 |
| NGIII | 2503.295 | 3639.361 | 1483.042 | 605.573 | 0.169 | 1.691 | 79.672* | 1045 |
| RBI | 2046.553 | 2978.178 | 1197.210 | 466.500 | 0.126 | 1.816 | 63.891* | 1045 |
| SCI | 1774.054 | 2432.615 | 1052.868 | 354.845 | 0.028 | 1.657 | 78.748* | 1045 |
| S&P 500 CEI | 471.200 | 623.460 | 289.140 | 80.703 | -0.012 | 1.782 | 64.664* | 1045 |
| S&PGLMCEI | 306.187 | 385.590 | 195.180 | 43.783 | 0.116 | 1.911 | 54.030* | 1045 |
| SII | 1852.339 | 2438.298 | 1208.236 | 376.003 | -0.112 | 1.564 | 92.012* | 1045 |
| WEGSLI | 221.527 | 291.444 | 137.904 | 37.963 | 0.082 | 1.774 | 66.702* | 1045 |
| WLCLI | 2680.897 | 3475.130 | 1658.789 | 445.961 | 0.054 | 1.764 | 67.065* | 1045 |
| WLCTI | 2650.860 | 3432.608 | 1653.070 | 439.257 | 0.052 | 1.765 | 66.978* | 1045 |

**Table 2. Unconditional correlations**

|  | RBI | NGIII | INI | SCI | FII | DEI | CSI | ATIII | WEGSLI | WLCTI | WLCLI | SII | S&PGLMCEI | S&P500CEI |
| --- | --- | --- | --- | --- | --- | --- | --- | --- | --- | --- | --- | --- | --- | --- |
| RBI |  |  |  |  |  |  |  |  |  |  |  |  |  |  |
| NGIII | 0.9075 |  |  |  |  |  |  |  |  |  |  |  |  |  |
| INI | 0.9048 | 0.9746 |  |  |  |  |  |  |  |  |  |  |  |  |
| SCI | 0.9686 | 0.8824 | 0.8778 |  |  |  |  |  |  |  |  |  |  |  |
| FII | 0.8928 | 0.9724 | 0.9691 | 0.8753 |  |  |  |  |  |  |  |  |  |  |
| DEI | 0.9113 | 0.9914 | 0.9689 | 0.8890 | 0.9756 |  |  |  |  |  |  |  |  |  |
| CSI | 0.8036 | 0.8666 | 0.8711 | 0.7870 | 0.8503 | 0.8636 |  |  |  |  |  |  |  |  |
| ATIII | 0.9436 | 0.9767 | 0.9615 | 0.9190 | 0.9466 | 0.9635 | 0.8462 |  |  |  |  |  |  |  |
| WEGSLI | 0.1278 | 0.1093 | 0.1072 | 0.1579 | 0.1160 | 0.1038 | 0.0724 | 0.1138 |  |  |  |  |  |  |
| WLCTI | 0.1295 | 0.1106 | 0.1091 | 0.1599 | 0.1182 | 0.1053 | 0.0743 | 0.1152 | 0.9962 |  |  |  |  |  |
| WLCLI | 0.1278 | 0.1090 | 0.1076 | 0.1584 | 0.1166 | 0.1040 | 0.0730 | 0.1136 | 0.9960 | 0.9997 |  |  |  |  |
| SII | 0.1132 | 0.0921 | 0.0936 | 0.1392 | 0.1042 | 0.0902 | 0.0540 | 0.1010 | 0.8348 | 0.8333 | 0.8339 |  |  |  |
| S&PGLMCEI | 0.1268 | 0.1087 | 0.1076 | 0.1579 | 0.1160 | 0.1043 | 0.0725 | 0.1125 | 0.9914 | 0.9958 | 0.9963 | 0.8532 |  |  |
| S&P500CEI | -0.0133 | 0.0031 | 0.0013 | -0.0165 | 0.0062 | 0.0072 | -0.0005 | -0.0047 | -0.0179 | -0.0198 | -0.0204 | -0.0310 | -0.0181 |  |
